# Ambivalence toward euthanasia and physician-assisted suicide has decreased among physicians in Finland

**DOI:** 10.1186/s12910-022-00810-y

**Published:** 2022-07-11

**Authors:** Reetta P. Piili, Pekka Louhiala, Jukka Vänskä, Juho T. Lehto

**Affiliations:** 1 Faculty of Medicine and Health Technology, Tampere University, Tampere, Finland; 2 Palliative Care Centre, Department of Oncology, Tampere University Hospital, Palliative Care Unit, Sädetie 6, R-building, 33520 Tampere, Finland; 3 Faculty of Social Sciences, Tampere University, Tampere, Finland; 4 Finnish Medical Association, Helsinki, Finland

**Keywords:** Euthanasia, Physician-assisted suicide, Physician

## Abstract

**Background:**

Debates around euthanasia and physician-assisted suicide (PAS) are ongoing around the globe. Public support has been mounting in Western countries, while some decline has been observed in the USA and Eastern Europe. Physicians’ support for euthanasia and PAS has been lower than that of the general public, but a trend toward higher acceptance among physicians has been seen in recent years. The aim of this study was to examine the current attitudes of Finnish physicians toward euthanasia and PAS and whether there have been changes in these attitudes over three decades.

**Methods:**

A questionnaire survey was conducted with all Finnish physicians of working age in 2020 and the results were compared to previous studies conducted in 1993, 2003 and 2013.

**Results:**

The proportions of physicians fully agreeing and fully disagreeing with the legalization of euthanasia increased from 1993 to 2020 (from 5 to 25%, *p* < 0.001, and from 30 to 34%, *p* < 0.001, respectively). The number of physicians, who expressed no opinion for or against euthanasia (cannot say) decreased from 19 to 5% (*p* < 0.001) during the same period. The proportion of physicians having no opinion (cannot say) of whether a physician should be punished for assisting in a suicide decreased from 20 to 10% (*p* < 0.001).

**Conclusions:**

This study shows that Finnish physicians’ ambivalence toward euthanasia and PAS has decreased. The ongoing debate has probably forced physicians to form more solid opinions on these matters. Our study highlights that attitudes toward euthanasia and PAS are still divided within the medical profession.

**Supplementary Information:**

The online version contains supplementary material available at 10.1186/s12910-022-00810-y.

## Introduction

The word euthanasia (εὐθανασία) is a combination of two Greek words: eu (εὖ), meaning well or good, and thanatos (θάνατος), meaning death. Thus, literally and etymologically euthanasia means “good death”.

Worldwide, euthanasia has been legalized in the Netherlands, Belgium, Luxembourg, Canada, the states of Victoria and Western Australia in Australia, New Zealand and Spain [1, 2]. In addition to these countries, PAS is legal in Switzerland, and in eight states in the USA (Maine, New Jersey, Oregon, Washington, Montana (court ruling), Vermont, Colorado, Hawaii and California) and in the District of Columbia [1, 2]. In Switzerland, assisted suicide is also available for Swiss nonresidents [1, 2]. In Columbia, there is a court ruling that physicians are not to be prosecuted for euthanasia or PAS, and Germany decriminalized assisted suicide in 2021 [1, 2]. In addition, debates about the legalization of euthanasia or physician-assisted suicide (PAS) are ongoing in many countries, including Italy, Portugal, Sweden and Finland. Although slowly spreading, assisted death is still a marginal phenomenon globally [3].

Public support for euthanasia and PAS is mounting across Western Europe, while some decline has been observed in the USA and Eastern Europe[4]. In Finland, different national studies show that public acceptance toward euthanasia increased from 50 to 85% between 1998 and 2017, although the exact wording of the questions was slightly different [5, 6]. In addition to discussions about assisted death, the importance of palliative care has been recognized among health care practitioners and the general public, and actions toward equal and high-quality palliative care have been taken in Finland.

Compared to the general public, a lower amount of support from physicians for euthanasia and PAS has been demonstrated in several surveys [4]. The variation in attitudes toward assisted death among physicians is large. A majority of physicians from Belgium and the Netherlands consider euthanasia and PAS to be justified in certain situations, whereas in Italy, only 36% of physicians were in favor of euthanasia in 2018 [4, 7]. In a recent study from Sweden, a clear trend toward more accepting attitudes was seen, as 47% of physicians accepted PAS in 2020 compared to 35% in 2007 [8]. Additionally, studies conducted among Finnish physicians show increased acceptability of euthanasia during the past 10 to 20 years, while attitudes toward PAS have not changed so radically [9–11].

Although attitudes toward assisted death among the public and physicians are becoming more permissible, the World Medical Association (WMA) considers euthanasia unethical [12]. The Finnish Medical Association is in line with the WMA and objects to the legalization of euthanasia [13]. Both the International Association for Hospice and Palliative Care (IAHPC) and the European Association for Palliative Care (EAPC) have quite recently stated that euthanasia and PAS should not be included as part of the clinical practice of palliative care [14, 15]. These statements are based on the idea that euthanasia and PAS contradict the main ethical principles. These main ethical principles include nonmaleficence (doing no harm), beneficence (doing good), autonomy (the right of self-determination), which often is the main argument for favoring assisted death, and justice (e.g. appropriate use and allocation of health care resources), as well as respecting life, which can be considered one of the fundamental principles of medicine [16, 17]. However, the British Medical Association recently adopted a neutral position on physician-assisted dying, and the Royal Dutch Medical Association is in line with local legislation, according to which physicians are allowed to perform euthanasia and PAS [18, 19]. These recent statements highlight the changing atmosphere toward assisted death, even among medical professionals.

Attitudes toward euthanasia and PAS have previously been studied in questionnaire surveys conducted in 1993, 2003 and 2013 among Finnish physicians. In 1993, 2003, and 2013, the questionnaire was sent to a random sample (n = 500, 840, 1003, respectively) of Finnish physicians of working age. Details of these three surveys can be found in our previous publications [8, 10].

The aim of this study was to reveal the current attitudes of Finnish physicians toward euthanasia and PAS and whether there have been changes in these attitudes over three decades, considering the increasing demands for the legalization of euthanasia among the Finnish public and the higher acceptance of assisted death among physicians in other countries. It was also important for the Finnish Medical Association to update its stance toward assisted death.

## Material and methods

### Participants

In this 2020 study, the survey was sent by email to all Finnish physicians and medical students who are members of the Finnish Medical Association and whose email address was available (n = 26,740). For this study, only physicians of working age (under 65 years old, not students) were included (n = 19,433). Two reminders were sent to nonresponders.

### Questionnaire

In all of the surveys, the questionnaire included five identical (except “and Belgium” was added to the second statement after 1993) statements about euthanasia and PAS: 1) euthanasia should be legalized in Finland, 2) a practice similar to that in the Netherlands and Belgium should be adopted, 3) a physician should be punished for assisting in a suicide, 4) with adequate terminal care and pain control, there is no need for (active) euthanasia, and 5) accepting (active) euthanasia would harm the doctor–patient relationship in general. (The word “active” was removed from the questionnaire in 2020 because it is outdated). In the questionnaire, PAS was defined as follows: a physician deliberately helping a person to commit suicide by giving drugs to the person to take them by him/herself by this person’s voluntary and competent request. Euthanasia was defined as follows: a physician deliberately killing a patient by administering drugs by the patient’s voluntary and competent request. The respondents were asked to express their agreement on these statements on a 5-point Likert scale, from fully agree to fully disagree or cannot say. In addition, some background information, such as age (in 1993, data not available), gender and self-reported experience in the care of dying patients (yes or no) or a request for assisted death from a patient or a relative, were asked. In 2020, the whole questionnaire included some broader aspects of assisted death and detailed issues on background factors, but these were not evaluated in this study. See the questionnaire from 2020 in the Additional file 1: Euthanasia and physicians-assisted death in 2020—a questionnaire for physicians.

### Ethical considerations.

The surveys were performed through the member registry of the Finnish Medical Association. The association has a permission to send questionnaires to its members if they have not declined this. Responding to the questionnaire was anonymous, and participation was voluntary. The data were anonymous when collected and analyzed. Participants gave their consent by voluntarily answering the questionnaire. The anonymous research data did not include any personally identifiable data. Therefore, we complied with national law and did not ask for prior consent to participate in the study prior [20]. According to the Finnish legislation, ethics approval is not needed in this type of questionnaire study [20]. This study was conducted according to national laws, regulations, and the Declaration of Helsinki.

### Statistical analysis

The responders and their answers are described with numbers and proportions. To assess different background factors on the answers concerning the statements “euthanasia should be legalized in Finland” and “a physician should be punished for assisting in a suicide”, the 5-point Likert scale was converted to two options: fully/partly agree and fully/partly disagree or cannot say (Table 3). These two-scale answers and background factors were tested using the Pearson chi-square test. Two-sided p values less than 0.05 were considered statistically significant. Data analysis was performed using IBM SPSS Statistics for Windows, Version 27.0 (IBM Corporation, 2020).

## Results

The characteristics of the responders are presented in Table 1. The response rates of the random samples in 1993, 2003 and 2013 were 73%, 57% and 48%, respectively. The response rate in 2020 from all Finnish working-age physicians was 24%. The number of female responders rose from 50% in 1993 to 65% in 2020. Slightly over half (54%) of the responders were over 45 years old, and approximately half of them were participating in the care of dying patients. Less than one-fifth had faced a question about euthanasia or PAS from a patient or relative.

**Table 1:** Characteristics of the respondents

	1993		2003		2013		2020	
Number (% of total)	365	(6)	479	(8)	481	(8)	4682	(78)
Response rate	73%		57%		48%		24%	
Female, n (%)	177	(50)	254	(54)	306	(64)	3027	(65)
Age distribution, n (%)*								
< 45 y	na		142	(30)	194	(41)	2263	(48)
≥ 45 y	na		331	(70)	285	(60)	2419	(52)
*Participates in the care of dying patients, n (%)*								
Yes	197	(56)	226	(48)	200	(42)	2419	(52)
No	157	(44)	248	(52)	274	(58)	2248	(48)
*Patient or relative having ask for euthanasia or PAS, n (%)*								
Yes	59	(17)	57	(12)	58	(12)	762	(16)
No	293	(83)	420	(88)	417	(88)	3920	(84)

^*^From 1993 data was not available

Table 2 presents the results concerning the statements on euthanasia and PAS according to the years of the surveys. The proportion of physicians fully agreeing with the legalization of euthanasia increased from 5% in 1993 to 25% in 2020 (*p* < 0.001), while the proportion of physicians having no opinion decreased from 19 to 5% during the same time period (*p* < 0.001). At the same time, the number of physicians fully disagreeing with the legalization of euthanasia rose from 30% in 1993 to 34% in 2020 (*p* < 0.001). The proportion of respondents fully disagreeing with the statement that a physician should be punished for assisting in a suicide increased from 14% in 1993 to 39% in 2020 (*p* < 0.001), and the proportion having no opinion on this decreased from 20% in 1993 to 10% in 2020 (*p* < 0.001). The proportions of responders who agreed with the statements on euthanasia and PAS according to the background factors are shown in Table 3. Responders who participated in the care of dying patients agreed with legalization less often and thought that physicians should be punished for assisted suicide more often than the others (42% vs. 49%, *p* < 0.001, and 28% vs. 24%, *p* < 0.001, respectively). When a patient or a relative asked for euthanasia or PAS, physicians supported the legalization of euthanasia more frequently (53% vs. 44%, *p* < 0.001), but there was no significant difference concerning PAS (24% vs. 26%, p = 0.114).

**Table 2:** Agreement with statements about euthanasia and PAS in 1993, 2003, 2013 and 2020

Statement	Fully agree				Partly agree				Partly disagree				Fully disagree				I cannot say			
	1993	2003	2013	2020	1993	2003	2013	2020	1993	2003	2013	2020	1993	2003	2013	2020	1993	2003	2013	2020
Euthanasia should be legalized in Finland	5%	9%	15%	25%	25%	21%	31%	24%	21%	16%	17%	13%	30%	46%	28%	34%	19%	9%	9%	5%
A practice similar to that in the Netherlands and Belgium should be adopted	12%	15%	24%	30%	37%	22%	28%	22%	14%	17%	15%	11%	23%	37%	26%	31%	13%	10%	7%	5%
A physician should be punished for assisting in a suicide	22%	14%	13%	11%	13%	18%	14%	13%	32%	28%	31%	27%	14%	22%	22%	39%	20%	18%	19%	10%
With adequate terminal care and pain control, there is no need for active* euthanasia	25%	32%	24%	31%	32%	31%	32%	26%	22%	23%	30%	27%	11%	9%	9%	14%	10%	6%	6%	2%
Accepting active*euthanasia would harm the doctor–patient relationship in general	22%	21%	14%	18%	26%	23%	19%	18%	23%	24%	27%	20%	15%	16%	21%	31%	15%	16%	20%	13%

^*^This word was removed from the questionnaire in 2020 due to the fact that it is outdated

**Table 3:** Proportions of responders (from all years) agreeing with the statements of euthanasia and PAS according to the background factors

	Euthanasia should be legalized in Finland		P-value*	A physician should be punished for assisting in a suicide		P-value*
	Fully/partly agree, n (%)			Fully/partly agree, n (%)		
Gender			< *0.001*			*0.002*
Female	1623	(43)		925	(25)	
Male	1060	(49)		611	(28)	
Age**		< *0.001*		0.904		
< 45 y	1263	(48)		660	(25)	
≥ 45 y	1345	(44)		775	(26)	
Participates in the care of dying patients, n (%)			< *0.001*			< *0.001*
Yes	1270	(42)		862	(28)	
No	1435	(49)		688	(24)	
Patient or relative having ask for euthanasia or PAS, n (%)			< *0.001*			0.114
Yes	493	(53)		225	(24)	
No	2223	(44)		1329	(26)	

Two -sided p-values < 0.05 were considered statistically significant

^*^ Pearson Chi-Square

^**^From 1993 data was not available

Responders who had faced a request for assisted death were more often males than females (18% vs. 14%, *p* < 0.001) and were involved in the care of dying patients (22% vs. 9%, *p* < 0.001), while this request was asked of 16% of the responders in both age groups (< 45 years and ≥ 45 years).

## Discussion

Based on the results of this study, Finnish physicians’ ambivalence toward euthanasia and PAS has decreased in recent years. The proportions of physicians fully agreeing and fully disagreeing with the legalization of euthanasia and fully disagreeing that a physician should be punished for assisting in a suicide have significantly increased, and the proportions of physicians being unsure of these matters have significantly decreased. The results from such a long follow up with identical questions have not been published from other countries, although there are surveys showing increased support for euthanasia and PAS from different countries across the globe [4, 8].

The reason why Finnish physicians’ attitudes on euthanasia or PAS have become less ambivalent is not clear. The increasing agreement for legalizing euthanasia among physicians found in our study might reflect the overall permissible atmosphere regarding assisted death in the general public. On the other hand, the number of physicians fully disagreeing with the legalization of euthanasia has been slightly rising, and the number of those who are unsure of their opinion has significantly decreased. During our study years, there has been an increasing public discussion of legalizing euthanasia in Finland. A citizen initiative demanding the legalization of euthanasia was raised in 2017 in Finland [21]. The Parliament rejected this initiative with clear numbers (129–59); during this process, the Social Affairs and Health Committee of the Parliament requested several expert opinions [22]. In 2018, the Finnish Ministry of Social Affairs and Health set up an expert group to consider legal options for euthanasia and end-of-life (EOL) care [23]. The National Advisory Board on Social Welfare and Health Care Ethics, ETENE, made a new statement on euthanasia in 2017, when it stated that it is not possible to estimate if there is a need for euthanasia as long as palliative care and hospice care are not sufficient everywhere in Finland [24]. At the same time, many other countries have decriminalized euthanasia since 1993 [1, 2]. In this context, euthanasia may no longer be seen as a theoretical question but as a reality and even a possible task in the future for Finnish physicians. This may force them to form a more solid opinion toward assisted death. However, we suggest that this polarization of opinions may further aggravate the already very complex debates around euthanasia in Finland. Actually, this has already been seen in Finland, as the expert group set several recommendations and improvements to legislation for palliative care and end-of-life care but gave only some suggestions without any clear recommendations about euthanasia or PAS in their statement in 2021 [23].

Attitudes toward PAS have more clearly moved in a direction where assistance in a suicide should not be punished. This is in line with previous studies from Finland and other countries showing more positive attitudes toward PAS [4, 8, 10]. Furthermore, taking someone else’s life is a punishable criminal act according to Finnish criminal law, which makes euthanasia illegal [25]. However, assisting in a suicide is not specifically stated as a criminal act in Finnish law, although this type of action, performed by a physician, has not been tested in a court of law in Finland, and a physician performing PAS might be prosecuted and convicted for a criminal deviation of good clinical practice. Another reason for more acceptable attitudes toward PAS might be the rise of individualism in Western countries, which is also seen in medicine [26]. Patients’ right to be involved in treatment decisions by shared decision-making and patient-centered care are today preferred by most physicians and patients [26–29]. These aspects may strengthen the tendency to accept the patient’s right to end his or her life and even enhance physicians’ willingness to help in this. However, an increased respect for the individual might also underlie a negative attitude toward legalizing assisted dying to protect the autonomy of the vulnerable and the dependent. Finally, there is probably a difference between demanding a punishment for a physician performing a PAS (asked in our study) and supporting the legalization of PAS, which must be taken into account when interpreting our results.

Answers to the statement that with adequate terminal care and pain control there is no need for (active) euthanasia have actually not changed much during the study years, as a little less than two-thirds of the responders agree fully or partly with this statement, regardless of the year of their answer. The number of responders answering that they fully disagree with the statement that accepting (active) euthanasia would harm the doctor–patient relationship has risen during the years studied, probably reflecting a more permissible atmosphere toward euthanasia also within the medical profession. However, over one third of the physicians still fully or partly agreed with this statement in 2020, highlighting the complexity of assisted death in doctor–patient relationship.

Differences between genders and age groups concerning euthanasia were statistically significant (Table 3), although the absolute differences in the proportions between the groups were rather low. Our findings are in line with previous studies in which younger physicians are more in favor of legalizing euthanasia [7, 30]. This difference was not seen in the statement concerning PAS, which differs from the study conducted in Sweden, where a more accepting attitude toward PAS among younger physicians was reported [8]. In a previous study from Finland, physicians considered PAS even more reprehensible than euthanasia, whereas in this study, only 24% of the responders considered that a physician should be punished for assisting in a suicide [10]. However, different wordings and ways of conducting surveys about euthanasia and PAS challenge the comparability of the results. Accepting PAS as a normal procedure in society may be regarded as different from specifically setting a legal punishment for a physician being somehow involved in the patient’s suicide (for example, by prescribing the drugs). It is known from previous studies that in the general population, men support the legalization of euthanasia more often than women, which is now seen in our findings from physicians as well [4]. Surprisingly, in our study, males also agreed more often with the statement that a physician should be punished for assisting in a suicide, which can be considered a quite conflicting result.

Approximately half of the responders reported participating in the care of dying patients. These physicians were significantly less in favor of legalizing euthanasia and more often thought that a physician should be punished for assisting in a suicide, although the absolute differences compared to other physicians were only moderate. Answering the question of whether a responder has participated in the care of dying patients was left without an explicit definition. Thus, physicians caring for dying patients only occasionally were probably included. This might have influenced our findings and the relatively high acceptance of legalizing euthanasia compared to earlier studies on physicians working in palliative care [8, 30, 31]. Nevertheless, our findings are in line with studies showing that the most experienced physicians in palliative care have the strongest opinions in their opposition to euthanasia and PAS [8, 30, 31]. What might explain this finding remains unknown. One reason could be that when adequate palliative care and high-quality symptom control are available, there is no need for euthanasia or PAS, which was agreed upon by more than half of the responders in our study. On the other hand, it could be argued that palliative care specialists also face the most difficult cases and deaths, where adequate symptom control may not be reached, which might lead to the conclusion that assisted death should be allowed. However, resolving inadequate symptom control with legalization of assisted death can be seen as a simplistic way of dealing with this ethically challenging dilemma. Another explanation for the hesitance of assisted death could be the fear of being forced to perform euthanasia and PAS in the near future against one’s own ethical principles, especially among the physicians working with palliative care. When a physician had faced a question from a patient or a relative regarding euthanasia or PAS, this significantly increased the agreement for the legalization of euthanasia in Finland. This kind of result was not reported previously, but it might be that physicians who already are in favor of euthanasia remember these requests better, or that seeing such suffering that makes a person ready to ask for euthanasia or PAS has influenced them in a way that these physicians think euthanasia/PAS could be a possibility to help the suffering. Another possible explanation might be the feeling of powerlessness in the physician, and this feeling might stem from the hopelessness of the patient’s situation. If a physician feels powerless in the face of a patient asking for euthanasia/PAS, this might also influence their attitudes toward assisted death in a more positive direction. The request for assisted death was more frequently asked from males and physicians caring for dying patients, of which the latter can be considered obvious, as the request is typically presented by a dying patient.

## Strengths and limitations

Limitations of this study need to be acknowledged. Our response rates are a limitation due to possible nonresponse bias. In contrast to the earlier ones, the 2020 study was conducted via an electronic platform and addressed to all Finnish physicians, resulting in a lower response rate but substantially more answers. Despite this, our study population is a representative sample of Finnish physicians, reflecting the changes in the medical profession, such as the rising numbers of female and young physicians [32, 33]. However, the methodologies of collecting the data differ between the present and earlier studies, and comparing the results must be done with caution. Nevertheless, we believe that comparisons can be made while keeping this limitation in mind. The questions concerning euthanasia and PAS were asked without giving explicit conditions under which these procedures were to be performed, and no details of the practices of assisted death in the Netherlands or Belgium were included in the questionnaire, which may have influenced the answers. Furthermore, questions concerning euthanasia (should it be legalized) and PAS (should a physician be punished for performing it) were presented in different ways, which prevented us from directly comparing these opinions. The time periods between the questionnaires were long, giving a unique overview of the attitudes and changes in these attitudes covering almost three decades. The electronic platform may have influenced the responses, but we believe the effect to be minimal, as the questions remained the same.

## Conclusions

Our study highlights that attitudes toward euthanasia and PAS are still controversial, dividing those within the medical profession. Attitudes have become stricter and less ambivalent, which probably reflects the increased discussion and higher awareness about euthanasia and PAS.

## Supplementary Information

**Additional file 1.** Euthanasia and physicians-assisted death in 2020—a questionnaire for physicians.

## Data Availability

The datasets generated and/or analyzed during the current study are not publicly available due to the current data policy of Finnish Medical Association but are available from the corresponding author on reasonable request.
